# Public health management of antiviral drugs during the 2009 H1N1 influenza pandemic: a survey of local health departments in California

**DOI:** 10.1186/1471-2458-12-82

**Published:** 2012-01-25

**Authors:** Jennifer C Hunter, Daniela C Rodríguez, Tomás J Aragón

**Affiliations:** 1 School of Public Health, University of California, Berkeley, CA, USA; 2 San Francisco Department of Public Health, San Francisco, CA, USA

**Keywords:** Public health preparedness and response, Public health systems research, Influenza

## Abstract

**Background:**

The large-scale deployment of antiviral drugs from the Strategic National Stockpile during the 2009 H1N1 influenza response provides a unique opportunity to study local public health implementation of the medical countermeasure dispensing capability in a prolonged event of national significance. This study aims to describe the range of methods used by local health departments (LHDs) in California to manage antiviral activities and to gain a better understanding of the related challenges experienced by health departments and their community partners.

**Methods:**

This research employed a mixed-methods approach. First, a multi-disciplinary focus group of pandemic influenza planners from key stakeholder groups in California was convened in order to generate ideas and identify critical themes related to the local implementation of antiviral activities during the H1N1 influenza response. These qualitative data informed the development of a web-based survey, which was distributed to all 61 LHDs in California for the purpose of assessing the experiences of a representative sample of local health agencies in a large region.

**Results:**

Forty-four LHDs participated in this study, representing 72% of the local public health agencies in California. While most communities dispensed a modest number of publicly purchased antivirals, LHDs nevertheless drew on their previous work and engaged in a number of antiviral activities, including: acquiring, allocating, distributing, dispensing, tracking, developing guidance, and communicating to the public and clinical community. LHDs also identified specific antiviral challenges presented by the H1N1 pandemic, including: reconciling multiple sources and versions of antiviral guidance, determining appropriate uses and recipients of publicly purchased antivirals, and staffing shortages.

**Conclusions:**

The 2009 H1N1 influenza pandemic presented an unusual opportunity to learn about the role of local public health in the management of antiviral response activities during a real public health emergency. Results of this study offer an important descriptive account of LHD management of publicly purchased antivirals, and provide practitioners, policy makers, and academics with a practice-based assessment of these events. The issues raised and the challenges faced by LHDs should be leveraged to inform public health planning for future pandemics and other emergency events that require medical countermeasure dispensing activities.

## Background

On April 26, 2009, the United States Government declared a public health emergency in response to the threat posed by the 2009 H1N1 influenza virus, A(H1N1)pdm09 [1]. This declaration triggered the Centers for Disease Control and Prevention (CDC) to ship large quantities of medical provisions from the Strategic National Stockpile (SNS) to state health departments around the nation in an effort to mitigate and control outbreaks of the novel virus. Included in this shipment were 11 million regimens of antiviral drugs (two neuraminidase inhibitors, oseltamivir and zanamivir), which were later accompanied by new federal guidance on the recommended clinical use of these drugs during the pandemic [2,3]. These events prompted state and local health departments to make decisions regarding how and where publicly purchased antivirals would be used in their communities to treat ill persons and slow the spread of disease.

The large-scale deployment of antivirals during the H1N1 influenza response presented a unique opportunity to study the local public health implementation of plans and protocols to support medical countermeasure dispensing. As one of the CDC public health emergency capabilities, *Medical Countermeasure Dispensing *is defined as, "the ability to provide medical countermeasures (including vaccines, antiviral drugs, antibiotics, antitoxin, etc.) in support of treatment or prophylaxis...to the identified population in accordance with public health guidelines and/or recommendations" [4]. The H1N1 influenza pandemic offered a highly unusual situation in which state and local health departments across the country simultaneously carried out this function during a prolonged event of national significance.

The need for the public health management of antiviral drugs during an influenza pandemic did not take public health officials by surprise. Prior to 2009, public health agencies and community partners had been actively engaged in preparedness activities in anticipation of antiviral utilization during an influenza pandemic. Among those efforts were large-scale purchase of antiviral drugs and the development of plans to appropriately use these medications to treat influenza illness and to reduce the impact of a pandemic [5]. However, given few opportunities to observe real-world response to an influenza pandemic, the preparedness community's understanding of state and local readiness for implementing a large-scale antiviral program has been limited. As a result, pre-pandemic assessments have reached wide-ranging conclusions regarding this preparedness capacity. In 2008, a federal assessment of pandemic influenza State Operating Plans found that "there are very few gaps in State readiness for antiviral drug distribution" [6]. Just one year later, the U.S. Department of Health and Human Services (HHS) Office of Inspector General reported notable gaps in antiviral plans at the local level, despite high scores in antiviral preparedness at the state level [7]. While these reports may, at first, seem contradictory, the findings illuminate the differences in the roles, responsibilities, and capacities of health departments at the state and local levels. For many states, including California, the primary responsibility for managing local antiviral drug activities resides with Local Health Departments (LHDs) in order to enable local control, planning, and implementation [8-10].

The 2009 H1N1 influenza response provided a valuable opportunity to learn from the experiences of LHDs during a real public health emergency. While the demand for antiviral drugs during the 2009 H1N1 influenza response was not as dramatic as had been predicted in many pre-pandemic planning scenarios [5,11], this paper seeks to illuminate how LHDs nevertheless drew on their previous work to meet the needs for antiviral drugs within their community. The H1N1 pandemic also tested antiviral planning assumptions and forced LHDs to creatively respond to new and unexpected challenges. The purpose of this study, then, is to describe the range of methods used by LHDs in California to manage antiviral activities and to gain a better understanding of the related challenges experienced by health departments and their community partners, with the goal of informing future planning for local public health preparedness and response efforts.

## Methods

This research employed a mixed-methods approach. First, a multi-disciplinary focus group of pandemic influenza planners from key stakeholder groups in California was convened in order to generate ideas and identify critical themes related to the local implementation of antiviral activities during the H1N1 influenza response. These qualitative data informed the development of a web-based survey, which was distributed to all 61 LHDs in California, for the purpose of assessing experiences of LHDs from a representative sample of local health agencies in California.

### Phase I - focus group

The first phase consisted of a teleconference-based focus group, comprised of members of the California Pandemic Influenza Vaccine and Antiviral (PIVA) Advisory Group. This statewide advisory group of pandemic planners and experts had convened prior to the 2009 H1N1 pandemic for the purpose of providing the California Department of Public Health with recommendations regarding vaccine and antiviral implementation prior to an influenza pandemic [12,13]. All PIVA Advisory Group members were invited to participate in the focus group via email, and were provided with an agenda that included an overview of focus group topics prior to the teleconference. The focus group topics included questions about antiviral acquisition, dispensing, and use. Also included were questions regarding challenges that were faced in these areas.

The focus group took place by phone in July 2010 and lasted 120 minutes. Twenty-three advisory group members participated in the teleconference, including representatives from state and local public health departments, federal agencies, private sector associations, academic institutions, hospitals, law enforcement agencies, and non-governmental organizations. The focus group was facilitated by one primary and one secondary facilitator. Notes were taken by the facilitators and an additional note taker, and were transcribed and merged into one document for review before being provided to focus group participants for participant validation. The focus group notes were reviewed to distill any relevant information that could be used to improve and strengthen the survey.

### Phase II - web-based survey

In the second phase of the study, a web-based survey was distributed to all 61 LHDs in California. This survey covered, in greater detail, the same antiviral topics discussed in the focus group informed by: focus group input, literature reviews, pre-pandemic planning with the PIVA Advisory Group, experiences of study personnel, and pilot testing with practice-based and academic-based experts. LHD involvement in the planning, coordination, and implementation of activities related to publicly purchased antivirals (i.e. those purchased by state or federal entities) were assessed in the following survey domains:

• Acquiring, distributing, allocating, dispensing, and use of antivirals;

• Tracking and monitoring the use of antivirals;

• Communications with other organizations regarding antivirals; and

• Challenges faced by LHDs in any of the aforementioned areas.

All LHDs within the state of California were recruited for participation in the survey, with the goal of obtaining a representative sample of LHDs' experiences managing antivirals during the H1N1 influenza response. A complete list of current LHD Health Officers and SNS Coordinators for each of the 61 LHDs in California (58 county health departments and 3 independent municipal health departments) was obtained from the California Department of Public Health. Based on the focus group discussion, persons in these two functional roles were expected to be most knowledgeable about the antiviral response at the local level.

The survey invitation was distributed to Health Officers and SNS Coordinators for all 61 LHDs along with a short description of the survey goals via email from the office of the Principal Investigator of Cal PREPARE, a CDC Preparedness and Emergency Response Research Center at the University of California, Berkeley. Recipients were instructed to forward the survey to the person within their department who was most informed about the health department's H1N1 influenza antiviral response and to submit one response for their health department. Survey data collection took place over the course of 3 weeks in August and September 2010. Two reminders were sent to invited participants via email in order to increase response rates.

Survey data were downloaded from the web survey provider and merged with county demographic data. Demographic data for the LHD catchment areas were collected from publicly available sources in order to classify LHDs by population size and to make comparisons of responding and non-responding agencies [14]. Health departments were classified into one of three population size categories based on the number of individuals served, adopting a categorization convention used by the National Association of County and City Health Officials: (1) fewer than 50,000 people, (2) between 50,000 and 499,999 people, and (3) 500,000 or more people [15]. Data were analyzed in Stata 11 (StataCorp LP, College Station, TX). A descriptive analysis of the survey results is presented here with quotations from the focus group and the survey to illustrate salient issues.

The protocol for this study was approved by the Committee for Protection of Human Subjects at the University of California, Berkeley.

## Results

These results describe the data obtained from LHD staff through the web-based survey.

### Sample demographics

Sixty-one LHDs were invited to participate in this study. Forty-four local health departments completed the survey, resulting in a 72% response rate. These counties represent 74% of the population of California. Participating agencies did not statistically differ from non-participating agencies with respect to the size of population served by the health department (chi-square with two degrees of freedom = 5.64, *p *= 0.35) or median household income (t = 0, *p *= 0.60). There was a borderline significant difference in the geographic distribution of responding counties (chi-square with two degrees of freedom = 5.64, *p *= 0.06), with the lowest participation in the southern region (46%) compared to the inland region (81%) and the coastal region (76%) [16].

The most widely represented functional role for respondents was SNS coordinators, with 55% identifying themselves as such. Other common functional roles were Emergency Preparedness Coordinator/Director and Health Officer with 25% and 27%, respectively (respondents could choose more than one functional role). Ninety eight percent of respondents stated that they were very knowledgeable about their LHD's antiviral activities during 2009 H1N1 response and 2% reported being somewhat knowledgeable about their LHD's response. Survey respondents who indicated that they knew only "a little" about their LHD's antiviral response were not allowed to complete the survey. Two respondents fell into this category and were not included in the overall response rate.

### Acquiring, distributing and allocating antivirals

#### Acquiring antivirals

All LHDs received antivirals from state or federal stockpiles, with a few LHDs receiving antivirals from a local cache or purchasing antivirals directly from the commercial or retail market as well. In this report, those antivirals purchased by state or federal government agencies for public use will be referred to as "publicly purchased antivirals" or "state and federal stockpiles."

#### Distributing antivirals

Twenty-eight LHDs reported distributing publicly purchased antivirals to other organizations for dispensing to the public, which accounts for 64% of respondents. The organizations and agencies that received antiviral drugs from LHDs are presented in Table 1. Among those most frequently cited were clinics, hospitals, and pharmacies (71%, 64%, and 54%, respectively). While many LHDs distributed antivirals to hospitals, others found that hospital pharmacies were unable to accept publicly purchased antivirals, as described by one participant,

**Table 1:** Number of LHDs that distributed antivirals to other organizations for dispensing to public (n = 28).

	Size of Population Served by LHD							
	**Small (< 50,000)**		**Medium (50,000-499,999)**		**Large (500,000+)**		**All LHDs**	

**Organization/Entity**	**n**	**(%)**	**n**	**(%)**	**n**	**(%)**	**n**	**(%)**

Public clinics/Health Centers	2	(50)	11	(69)	7	(88)	20	(71)

Private hospitals	0	(0)	10	(63)	8	(100)	18	(64)**

Retail pharmacies	3	(75)	8	(50)	4	(50)	15	(54)

Public Hospitals	2	(50)	3	(19)	8	(100)	13	(46)

Private clinicians	0	(0)	5	(31)	4	(50)	9	(32)

Tribal health clinic/hospitals	1	(25)	4	(25)	3	(38)	8	(29)

Prisons	0	(0)	1	(6)	4	(50)	5	(18)

College/Universities	0	(0)	4	(25)	1	(13)	5	(18)

Direct to patient*	0	(0)	3	(19)	2	(25)	5	(18)

Skilled nursing facilities/Long term care facilities	1	(25)	1	(6)	2	(25)	4	(14)

Military bases	0	(0)	0	(0)	0	(0)	0	(0)

Airports	0	(0)	0	(0)	0	(0)	0	(0)

Other	1	(25)	3	(19)	1	(13)	5	(18)

Total	4		16		8		28	

Among the twenty-eight local health departments (LHDs) that distributed publicly purchased antivirals to other organizations for dispensing to the public, Table 1 summarizes the number and proportion of LHDs that reported distributing antivirals to each type of organization or entity, stratified by size of population served by LHD

*Direct to patient methods include household delivery and pick-up and health department

**Indicates statistically significant difference with respect to population size served by LHD (Fisher's exact test, *p *< .05)

"We contracted with [chain retail pharmacy] for this service since hospitals could not accept public antivirals into their pharmacies."

Receiving organizations classified under "Other" include HIV care providers, addiction programs, homeless centers and children's homes.

#### Allocating antivirals

LHDs selected organizations to dispense publicly purchased antivirals for the following reasons: to reach the uninsured or underinsured (89%), to reach severely ill persons (61%), to reach medically at-risk persons such as pregnant women (57%), and/or because the organization was well-known in the community (61%). LHDs also noted that dispensing sites were chosen based on storage capacity or the ability of a pharmacy to correctly reconstitute antiviral suspension for children.

The number of antivirals allocated to these dispensing sites was determined based on the characteristics of the patient population served (41%), requests for antivirals (37%), number of persons served by a medical or treatment facility (33%), and/or epidemiologic data and patient volume (26%).

Eight of the 28 LHDs that distributed antivirals to other organizations for dispensing to the public reported that they required facilities to show that antivirals were not commercially available before they could receive publicly purchased antivirals (28%). This strategy was based on the rationale that publicly purchased antivirals should be used only as a last resort after the commercial and retail markets had been depleted.

### Antiviral dispensing, use and shortages

#### Antiviral dispensing

Thirty-one LHDs reported that publicly purchased antivirals were dispensed for patient use in their jurisdiction (70%). Of these LHDs, approximately 90% dispensed fewer than 250 doses of antivirals (ranging from less than 100 doses to more than 10,000 doses). On average, the reported number of antiviral doses dispensed in a community increased with population size. For small LHDs serving fewer than 50,000 residents, the modal number of antiviral doses dispensed was 0 doses (ranging from 0 to 250 doses); for medium LHDs serving a population of 50,000-499,999, the modal number of antiviral doses dispensed was less than 250 doses (ranging from 0 to 1,000 doses); and for large LHDs serving a population of 500,000 and more, the modal number was less than 250 doses (ranging from 0 to more than 10,000 doses). For those LHDs reporting that publicly stockpiled antivirals were not dispensed in their jurisdiction, many reported that they had few influenza cases and demand for antivirals was low. Others indicated that their strategy was to use publicly purchased antivirals when shortages were reported in the local commercial market, which did not occur in their communities.

All LHDs that distributed publicly purchased antivirals to dispensing sites required those organizations to agree to certain terms of dispensing. Eighty six percent of these 28 LHDs required dispensing sites to track antiviral utilization and report it to the health department. Providing antivirals free of charge to the uninsured or underinsured and providing antivirals free of charge to all were required by 61% and 50% of these LHDs, respectively. Other requirements noted by respondents included: returning unused antivirals to the LHD, following current recommendations for use, requesting documentation from recipients to ensure eligibility, and the use of temperature-controlled storage.

#### Antiviral use

Potential uses of antiviral drugs include treatment, pre-exposure prophylaxis, and post-exposure prophylaxis. LHDs were asked about allowable uses for publicly purchased antivirals in their community and the target groups for each allowable use (Tables 2, 3 and 4). In addition to the target groups presented in Tables 3 and 4, several LHDs further specified that restrictions on populations eligible for publicly purchased antivirals for treatment were lifted when antivirals were not commercially available. Regarding prophylaxis, LHDs noted that publicly purchased antivirals were dispensed to uninsured or underinsured persons, children, and household members of ill persons early in the pandemic in order to slow transmission of the virus.

**Table 2:** Allowable uses for publicly purchased antivirals (n = 31).

Allowable Use	n	(% of LHDs)
Treatment of ill persons	28	(90)

Post-Exposure Prophylaxis (PEP)	24	(77)

Pre-Exposure Prophylaxis (PrEP)	7	(23)

Allowable use determined by dispensing site	8	(26)

Other	2	(6)

Among the thirty-one local health departments (LHDs) reporting that publicly purchased antivirals were dispensed in their community, Table 2 summarizes the number and proportion of LHDs that allowed publicly purchased antivirals to be used for treatment, post-exposure prophylaxis, and pre-exposure prophylaxis

**Table 3:** Target groups for treatment with publicly purchased antivirals (n = 28).

	Size of Population Served by LHD							
	**Small (< 50,000)**		**Medium (50,000-499,999)**		**Large (500,000+)**		**All LHDs**	

**Eligible Groups**	**n**	**(%)**	**n**	**(%)**	**n**	**(%)**	**n**	**(%)**

Uninsured or underinsured persons	3	(75)	11	(69)	3	(38)	17	(61)

Any ill person	2	(50)	3	(19)	5	(63)	10	(36)

Persons at high-risk for medical complications of influenza (e.g. pregnant women)	1	(25)	5	(31)	1	(13)	7	(25)

Persons in an occupation-based target group (e.g. healthcare workers)	1	(25)	4	(25)	0	(0)	5	(18)

Other	0	(0)	3	(19)	1	(13)	4	(14)

Don't know/Unable to answer	0	(0)	0	(0)	0	(0)	0	(0)

Total	4		16		8		28	

Among the twenty-eight local health departments (LHDs) reporting that publicly purchased antivirals could be used for treatment, Table 3 summarizes the groups eligible for antiviral treatment with publicly purchased antivirals, stratified by size of population served by LHD

**Indicates statistically significant difference with respect to population size served by LHD (Fisher's exact test, *p *< .05)

**Table 4:** Target groups for prophylaxis with publicly purchased antivirals (n = 25).

	Size of Population Served							
	**Small (< 50,000)**		**Medium (50,000-499,999)**		**Large (500,000+)**		**All LHDs**	

**Eligible Group**	**n**	**(%)**	**n**	**(%)**	**n**	**(%)**	**n**	**(%)**

Household members of persons with H1N1 influenza	3	(100)	10	(77)	4	(44)	17	(68)

Persons at high-risk for medical complications of influenza (e.g. pregnant women)	3	(100)	4	(31)	5	(56)	12	(48)

Healthcare workers	2	(67)	4	(31)	3	(33)	9	(36)

Other first responders	2	(67)	1	(8)	1	(11)	4	(16)

Household members of healthcare workers	1	(33)	0	(0)	1	(11)	2	(8)

Other	0	(0)	3	(23)	3	(33)	6	(24)

Don't know/Unable to answer	0	(0)	2	(15)	2	(22)	4	(16)

Total	3		13		9		25	

Among the twenty-five local health departments (LHDs) reporting that publicly purchased antivirals could be used for pre- or post-prophylaxis, Table 4 summarizes the groups eligible for prophylaxis with publicly purchased antivirals, stratified by size of population served by LHD

**Indicates statistically significant difference with respect to population size served by LHD (Fisher's exact test, *p *< .05)

In order to determine eligibility to receive publicly purchased antivirals, 24 of the 31 LHDs reporting that publicly purchased antivirals were dispensed for patient use in their jurisdiction instituted verification procedures. Fifty four percent of theses LHDs reported using pharmacists or dispensing sites to determine eligibility status, 46% reported that physicians provided proof of eligibility, and 25% indicated that patients self-reported their eligibility status. Six LHDs did not require any eligibility verification.

#### Antiviral shortages

Among all LHD respondents, 26 indicated that their communities experienced shortages of at least one type of antiviral drug (60%). Shortages were primarily of pediatric formulation of oseltamivir, followed by adult formulation of oseltamivir (reported by 96% and 38% of LHDs with shortages, respectively). No shortages of zanamivir or peramivir were reported.

### Antiviral tracking and monitoring

LHDs were asked about their ability to track the movement of antivirals from stockpile sites to recipients. Of the 28 health departments that distributed antivirals, nearly all were able to track the federal/state stockpile to their health department (93%), and from the health department to dispensing sites (100%). Most LHDs were able to track from dispensing sites to recipients (71%).

Twenty-four LHDs received antiviral utilization data from dispensing sites with varying degrees of frequency. Half of these LHDs received antiviral utilization reports weekly or more frequently. Some health departments received basic utilization data, such as number of courses dispensed or lot numbers, while others received more specific data, including demographic information, intended use of antivirals (e.g. treatment, PEP), or reason that public stockpiles were utilized (e.g. shortages, uninsured recipient). These data were primarily used for making allocation decisions, surveillance, future antiviral planning, reaching target populations, and providing data to other agencies. Utilization data were received primarily by fax, but also by email, phone calls, face-to-face meetings, and mail.

### Communications

The most common mechanisms LHDs used for communicating with other organizations participating in the antiviral response were to following: email (56%), blast-fax (53%), phone (49%), and in-person/face-to-face meetings (42%). Other commonly cited communications mechanisms were teleconference (33%), websites (30%), and the California Health Alert Network, known as CAHAN (21%). The remaining LHDs indicated that communication with other agencies around antivirals did not occur in their jurisdiction or was not applicable to their health department's response.

LHDs also used various mechanisms to inform the public about where eligible persons could obtain antivirals. Around half of LHDs indicated that the health department (57%) or the clinician (45%) were the source of information for these individuals. Others relied on pharmacists (32%), the media (16%), or employers (7%). The remaining LHDs did not communicate with the public because they perceived antiviral need to be low.

Only six LHDs found that there were stakeholder groups with whom they had to establish communications for the antiviral response that had not been originally planned. These stakeholder groups included: smaller clinics, retail pharmacies, certain minority populations, community clinics, local school districts, community based organizations, state correctional facilities, and indigent populations.

### Antiviral challenges for LHDs

**Figure 1:**
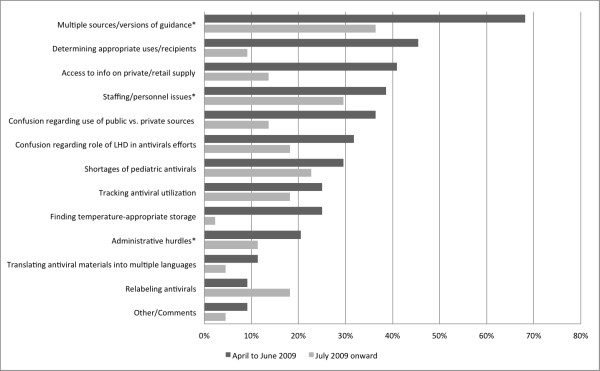
**Antiviral challenges reported by Local Health Departments, by time period (n = 44)**. Proportion of LHDs reporting each antiviral challenge, by time period (April to June 2009 and June 2009 onward). Among those LHDs reporting a challenge, those issues reported to be "very challenging" by 40% or more respondents are marked by an asterisk.

LHDs were asked about antiviral challenges faced and feedback received during the H1N1 influenza response. In terms of challenges, LHDs were asked to identify issues that were problematic early and later during the pandemic response. "Early" was defined as April to June 2009 and "Later" was defined as July 2009 onward. These challenges are presented in Figure 1.

Changes in antiviral guidance and multiple sources of information about antivirals presented a serious challenge to LHDs. This was the most widely cited issue both early and late in the pandemic, and was described as 'very challenging' by more than a third of health departments. One LHD noted, *"Changing guidance made it difficult to determine appropriate recipients." *Other participants indicated that state and federal antiviral guidance during H1N1 differed from what they had anticipated, particularly with respect to the target population for stockpiled antivirals. This discrepancy required their health departments to revise their planned activities to support this strategy. As several respondents indicated,

"The drugs from the SNS were originally intended to [be] used only when the drugs were no longer available through the commercial market. During the H1N1 epidemic the state required that we make the drugs available to people who were unable to afford the meds."

"The state's late decision to make the SNS antivirals available on a compassionate basis...created a great deal of trouble for my department."

"....had this event been more widespread, guidance from the state could have caused considerable issues related to the first responder community."

Shortages of pediatric antivirals and staffing issues also remained significant challenges for LHDs throughout the pandemic. The staffing issues were highlighted by one respondent,

"H1N1 served as a 'dry run' for a more severe pandemic. Had we been faced with the need to distribute antiviral drugs to multiple health care and other venues, to a large percentage of our population, over a prolonged period of time, we would have been severely challenged to do so in a secure, accountable, consistent, and equitable manner. Given decreasing public health resources, I do not foresee an improvement in our capacity to do this anytime in the near future."

Early in the pandemic, the main challenges centered on ramping up response efforts, such as: determining uses and target groups for antivirals, ascertaining information about antiviral availability in the commercial market, finding temperature-appropriate storage, and managing confusion about the use of public versus private stockpile antivirals. All challenges were cited less frequently during the later time period, with the exception of relabeling antivirals, which was mentioned twice as frequently in the later time period.

For those LHDs experiencing each challenge, the issues most likely to be reported as 'very challenging' were staffing and personnel issues, changing antiviral guidance and multiple sources of information, and administrative hurdles (these "very challenging" issues are marked with an asterisk in Figure 1). An example of the type of administrative hurdles faced by LHDs was illustrated by one respondent,

"We [the LHD] were advised [by the county] ...that the county would need to consider rapid purchase of antivirals for protection of essential county workers and possibly health care workers. We were asked to develop a special agreement to have local pharmacies make the purchase investment and guarantee them employee utilization and billing of insurance. At the start of the pandemic, this additional administrative task to avoid a sizeable county expenditure in antivirals for ongoing prophylaxis was an added task for our small health department."

#### Feedback from community partners

LHDs reported receiving negative feedback from community partners regarding a number of issues related to antivirals (Table 5). The most frequently mentioned issues centered on confusion around appropriate uses and target groups for antivirals and especially publicly purchased antivirals.

**Table 5:** Negative feedback received by LHDs from community partners (n = 44)

Feedback	n	(% of LHDs)
Confusion regarding clinical guidance for antiviral use	18	(41)

Confusion regarding the use of public vs. private stockpile antivirals	11	(25)

Dissatisfaction regarding which patients should receive antivirals from public stockpile	11	(25)

Antiviral availability	10	(23)**

Administrative challenges associated with antivirals	10	(23)

Burdensome paperwork associated with tracking antiviral utilization	4	(9)

Difficulty determining whether local stockpiles had been depleted	3	(7)

Reported misuse of publicly purchased antivirals	2	(5)

Antiviral storage or security	1	(2)

**Indicates statistically significant difference with respect to population size served by LHD (Fisher's exact test, *p *< .05)

Private clinicians and hospital/healthcare facilities were the most frequently cited sources of negative feedback, reported by 30% and 16% of LHDs respectively. LHDs that received negative feedback from these entities commonly also reported negative feedback in the following areas: confusion regarding clinical guidance for antiviral use, dissatisfaction regarding which patients should receive publicly purchased antivirals, and confusion regarding the use of the public versus private sources of antivirals. Medium sized health departments (those serving populations between 50,000-499,999 persons) were more likely to report hearing negative feedback regarding antiviral availability (Fisher's Exact test, p < 0.05).

Only five LHDs reported difficulties or concerns in providing antivirals to specific populations. The populations noted by respondents included homeless/transient populations, undocumented persons, and pregnant women. One respondent described how reimbursement issues in Medi-Cal (California's state Medicaid insurance program for the poor and underserved) made it difficult to reach pregnant women early in the pandemic,

"...We had issues in the beginning with Medi-Cal patients namely pregnant women that did not get access to the stockpiled antivirals because Medi-Cal took so long to change their formulary and a communication (or other) issue related to getting the women to a location with the antivirals existed. This was remedied when Medi-Cal changed their formulary."

LHDs also noted positive experiences working with the state health departments, including,

"The antiviral acquisition, allocation, distribution, and dispensing was probably the smoothest [part of the] response during the H1N1 campaign. State did a great job getting them out to the LHD's as well as retrieving them."

## Discussion

While recent research has demonstrated the clinical importance of antivirals during the H1N1 influenza response [17-19], the role public health agencies in the management of these drugs at the local level has not been well studied. To the best of our knowledge, this is the first study to examine the antiviral activities carried out by LHDs during the H1N1 influenza response using a representative sample from a large geographic area. A major finding of this report is that, while the number of publicly purchased antivirals dispensed was limited in most communities (fewer than 10% of LHDs reported that more than 250 courses were dispensed in their jurisdiction), LHDs nevertheless drew on their previous work and engaged in a number of antiviral activities. LHDs successfully coordinated with the state health department to receive antivirals from the SNS, made decisions regarding when and where these antivirals would be dispensed within their community, determined which groups would be eligible for these antivirals, allocated and distributed antivirals to dispensing sites for the purpose of reaching target groups, developed systems for verifying eligibility for antivirals and tracking antiviral utilization, and provided guidance to the clinical community.

This study also documents specific challenges presented by the H1N1 pandemic that were faced, and overcome, by LHDs. Our research corroborates and compliments a recent qualitative investigation on this topic, conducted by National Association of County and City Health Officials (NACCHO) researchers [20]. Using different methods, both studies find that LHDs had difficulty reconciling multiple sources and versions of antiviral guidance from state and federal agencies, and that this was a major challenge at the local level. This was not only the most commonly reported difficulty in our respondents; it was also one of the most likely to be characterized as "very challenging." NACCHO researchers aptly describe two potential contributors to confusion at the local level that appear to be supported by our findings. First, federal antiviral guidance during the H1N1 response primarily focused on clinical recommendations for antiviral use, and did not directly address how public stockpiles should be used. Second, clinical antiviral guidance changed during the course of the pandemic, which placed a greater emphasis on the use of antivirals for early treatment rather than post-exposure prophylaxis later in the pandemic [20].

Our research also demonstrates that the recommended uses and recipients of publicly purchased antivirals during the H1N1 response differed from what LHDs had anticipated in their pre-pandemic plans, and that this resulted in additional difficulties. During the 2009 H1N1 pandemic, antivirals were generally available through normal wholesale and retail markets; as a result, the State health department recommended that LHDs use publicly purchased drugs for the treatment of uninsured or underinsured persons and for communities experiencing shortages [3,21]. For many health departments, this represented a significant shift in their pre-pandemic antiviral implementation strategy, which had focused on the use of publicly purchased antivirals for treatment of ill persons once retail supplies had been depleted and that had emphasized the role of antivirals in protecting healthcare workers and other first responders [4]. As a consequence, LHDs revised their plans to support these new strategies, though implementing and communicating these strategies caused a strain on some local health departments and community partners.

Another outcome with particular relevance to public health planning is the effect of staffing and personnel shortages on the public health antiviral response. Among the nineteen agencies that cited staffing issues as a challenge during their antiviral response, nearly half found this to be "very challenging." These findings are consistent with the documented workforce reductions in local public health since 2008 [22]. As noted by one participant, current public health resources are insufficient for local health agencies to confidently deliver antiviral services in a *"secure, accountable, consistent, and equitable manner" *during a pandemic with a larger scope or greater severity. These concerns should be taken into account in the future development of antiviral and medical countermeasure plans and policies.

Public health systems researchers have noted that variations in availability of community resources and community preferences will influence how public health services will be implemented [23]. In this study we observed great variability in the approaches used by LHD to manage publicly purchased antiviral drugs. It is expected that some of this variability is attributable to differences in the circumstances faced by LHDs (e.g. influenza illness incidence, community demographics, availability of antivirals in the retail market) while other variation is due to differences in disease control and prevention strategies. It is beyond the scope of this study to evaluate the effectiveness of different approaches; however, the findings may inform preparedness conversations regarding which variations in practice are seen as beneficial and adaptive and which areas might benefit from uniformity. State and local health departments, in California and elsewhere, can study these variations in practice and select models and promising practices to be included in their response planning.

Lastly, this report builds upon the Association of State and Territorial Health Officials (ASTHO) assessment of early experiences of state and territorial health departments in receiving and distributing antivirals from the SNS [24]. Because state and local public health agencies serve different roles and functions in the antiviral response, there is a demonstrated need to document activities at both levels [10,25,26]. Whereas the ASTHO research describes how publicly purchased antivirals were allocated and deployed from the SNS and then distributed and received at the state-level in California, our research tracks these antivirals into communities and further explicates how LHDs managed and directed this resource.

## Limitations

There are several limitations to this study. First, we chose to permit only one response per LHD and allowed anyone within the LHD to respond. Thus, we received responses from SNS coordinators, health officers and other LHD personnel. It is possible that SNS coordinators and other health department staff, especially health officers, experienced the 2009 H1N1 influenza pandemic differently given their different roles. To account for this, we gave LHDs the flexibility of choosing the person who was most familiar with their antiviral response. Given that ninety eight percent of respondents stated that they knew "a lot" about their agency's antiviral response activities, we consider their perspectives to be a valid representation of the LHDs' experience.

A second limitation is the number of LHDs represented in the study. While this sample includes health departments that represent nearly three-quarters of the 40 million residents in California - our unit of analysis is the health department, resulting in a small absolute number of cases. Furthermore, the communities served by these LHDs differ dramatically with respect to organizational and demographic factors. For example, the smallest health department in California represents 1,200 individuals and the largest serves over 8 million. Because of these differences, we present some of our results stratified by the size of the population served by the LHD. However, once the data are divided by population size (or any other community characteristics of interest), the number of health departments represented in each stratum is very small, recommending caution in making strong inferences about any of the observed differences.

Lastly, because this research focused on LHDs in California, it is possible that the findings are unique to this state. However, given concurrence of our research findings with previous qualitative work with a wider geographic scope [20], we believe these results are more broadly applicable beyond California. To improve our understanding of the role of LHDs in the management of antivirals, this work should be replicated in a state with a different organizational, political, or authority structure is in place, which might be expected to contribute to different experiences at the local level (e.g. a large state with a centralized public health authority) [27]. Additional studies from other nations that also used publicly purchased antivirals during the H1N1 influenza response, particularly those with different policies on antiviral drug use, would cast important light on this issue.

## Conclusion

Prior to 2009, state and federal agencies made substantial investments in antiviral drugs for use during a influenza pandemic, including the purchase of nearly 81 million courses of antiviral drugs. The large-scale deployment of this asset during the H1N1 influenza response provides us unique learning opportunity regarding the public health role in the management of antiviral activities at the local level, an area of the H1N1 influenza response that has not been well studied. Results of this study therefore offer an important descriptive account of LHD management of publicly purchased antivirals in California, and provide practitioners, policy makers, and academics with a practice-based assessment of these events. The issues raised and the challenges faced by LHDs during H1N1 should be leveraged to inform public health planning for future pandemics and other emergency events that require medical countermeasure dispensing activities.

## Competing interests

The authors declare that they have no competing interests.

## Authors' contributions

JH and TA conceived of the study. JH and DR coordinated and implemented study recruitment and data collection. All authors participated in the design of the study, development of data collection tools, analyzing and interpreting the data, and helped to draft the manuscript. All authors read and approved the final manuscript.

## Pre-publication history

The pre-publication history for this paper can be accessed here:

http://www.biomedcentral.com/1471-2458/12/82/prepub
